# Development of performance indicators for systems of urgent and emergency care in the Republic of Ireland. Update of a systematic review and consensus development exercise

**DOI:** 10.12688/hrbopenres.12805.2

**Published:** 2019-02-12

**Authors:** Siobhan Boyle, Rebecca Dennehy, Orla Healy, John Browne

**Affiliations:** 1 School of Public Health, University College Cork, Cork, Ireland; 2 Department of Public Health, Health Service Executive, Dublin, Ireland

**Keywords:** urgent and emergency care systems, performance indicators, accident & emergency medicine, consensus development group exercise, urgent care-sensitive conditions, serious emergency conditions

## Abstract

**Objectives: **To develop a set of performance indicators to monitor the performance of emergency and urgent care systems in the Republic of Ireland.

**Design**: This study comprised of an update of a previously performed systematic review and a formal consensus development exercise. The literature search was conducted in PubMed and covered the period 2008 to 2014. The results of the review were used to inform a consensus group of 17 national experts on urgent and emergency care in Ireland. The consensus development exercise comprised an online survey followed by a face-to-face nominal group technique meeting. During this meeting participants had the opportunity to revise their preferences for different indicators after listening to the views of other group members. A final online survey was then used to confirm the preferences of participants.

**Results: **Initial literature searches yielded 2339 article titles.  After further searches, sixty items were identified for full-text review. Following this review, fifty-seven were excluded. Three articles were identified for inclusion in the systematic review. These papers produced 42 unique indicators for consideration during the consensus development exercise. In total, 17 indicators had a median of greater than 7 following the meeting and met our pre-specified criterion for acceptable consensus.

**Discussion: **Using this systematic review and nominal group consensus development exercise, we have identified a set of 17 indicators, which a consensus of different experts regard as potentially good measures of the performance of urgent and emergency care systems in Ireland. Pragmatic implications are discussed with reference to three subsequently performed original studies which used some of the indicators

## Introduction

Emergency and urgent care consists of all the services which contribute to the management of people when immediate care is sought for a health condition. When patients need immediate care they can enter the health system through a range of services and will often use more than one. This can lead to a duplication of services, confusion about the most appropriate access point for individual patients and the danger of poorly co-ordinated care, especially at the point where patients transfer from one service to another. Emergency and urgent care services include pharmacy, primary care, minor injury units, acute medical assessment units, emergency departments, mental health services and all the services required to refer and transport patients to an appropriate treatment facility.

There is an increasing awareness that urgent and emergency care services should operate as whole systems of care for the populations they serve ^1^. Adopting such an approach requires individual service providers to be integrated into larger systems and to co-ordinate their activities accordingly. It is hoped that a systems approach will deliver a higher standard of quality, safety, efficiency, timeliness and overall patient experience without introducing inequity of access. Policy makers have a variety of tools at their disposal when attempting to engineer a systems approach to urgent and emergency care. These include the centralisation of care for high risk cases at high volume hospital units and the use of referral pathways and new facilities such as minor injury units to direct low-risk cases to settings that are appropriate for their condition ^2^. Other elements include the use of telemedicine to provide support to smaller facilities and the development of community services for patients with conditions that are sensitive to the quality of ambulatory care ^3^.

The Health Service Executive (HSE) is responsible for the provision of publically funded health services in the Republic of Ireland. The HSE has attempted to foster a systems approach to urgent and emergency care services across the whole country, but the pace and nature of change is highly variable. In four peripheral regions (South, West, Mid-West, North-East) the reconfiguration process is at an advanced stage, but progress has been much slower in Dublin, the Midlands and the South-Eastern part of the country. This variation represents a natural experiment in policy making and is an opportunity to observe the impact of the changes that have been introduced before they are implemented across the whole country.

Existing indicators of urgent and emergency care performance focus on individual services and do not capture the performance of systems ^4^. The development of such indicators would allow policy makers to compare different models of care and evaluate the longitudinal impact of changes to service configuration. In light of this and considering the introduction of a system-based approach to urgent and emergency care by the HSE, the aim of this study was to develop a set of performance indicators to monitor the performance of emergency and urgent care systems in the Republic of Ireland.

## Methods

### Systematic review

This study comprised of an update of a previously performed systematic review which had covered the period up to 2007 ^5^ and a formal consensus development exercise. The systematic review update was conducted in August 2014 by one person (RD). Articles cited in PubMed over the period 2008 to 2014 were systematically searched by combining variations of the text terms ‘emergency’ and ‘indicator’ using the AND operator. Our search for novel indicators was supplemented by a review of the reference lists of articles selected for review. We also contacted experts and organisations working on the assessment of urgent and emergency care performance to identify relevant grey literature. These included the Society for Academic Emergency Medicine (USA), the Centre for Medicare and Medical Services (USA), the Emergency Department Benchmarking Alliance (USA), the Canadian Association of Emergency Physicians, the European Society for Emergency Medicine, the Royal College of Surgeons England, the Pre-Hospital Emergency Care Council (Ireland) and relevant HSE Clinical Programme Directorates.

Articles were selected for review by two persons (RD and JB) on the basis that they might contain definitions of system-level indicators of emergency and urgent care performance. Articles were excluded after review of the full text version if the indicators that they contained were already listed by the previous systematic review or if they focused on individual components of the urgent and emergency care system such as emergency department waiting times or ambulance response times. Non-English articles were also excluded. Included articles were read and discussed by RD and JB who came to a consensus about whether they contained at least one indicator that measured system-level performance in urgent and emergency care. A conservative approach was taken and indicators were excluded only if they clearly related to a specific service and did not capture elements of system function. This approach was warranted on the basis that the subsequent consensus development exercise was to be the definitive judgement on the extent to which indicators were useful signals of system performance.

The systematic review has been reported according to PRISMA ( Supplementary File 1).

### Consensus development exercise

The consensus development exercise comprised an online survey and a face-to-face nominal group meeting. A broad range of Irish-based experts were recruited to the consensus development group. Experts were recruited by contacting professional representative bodies, policy making organisations, regulatory bodies and patient advocacy groups. The following clinical disciplines were recruited to the group: emergency nursing, acute medicine, minor injuries/urgent care nursing, anaesthesia/intensive care, emergency medicine, psychiatry, public health, paediatrics, pre-hospital care, general practice, pharmacy and geriatric medicine. The HSE quality improvement directorate, the Irish Department of Health, the Irish healthcare regulator (Health Information and Quality Authority) and two patient advocacy groups were also represented. Once individuals were highlighted as potential members, they were approached through email and phone calls to join the group. Interested parties were then sent a formal invitation letter to join the group. In total the group was composed of 17 national experts on urgent and emergency care in Ireland.

All novel indicators identified in the updated systematic review were combined with those identified in the original systematic review and grouped under the following headings in an online survey: outcome based indicators, process based indicators and structural indicators (see Supplementary File 2). The definitions of urgent and emergency conditions were adopted from those used in previous consensus development work ^4^ performed by the University of Sheffield for the English NHS (see Supplementary File 3). The survey was designed and distributed to the consensus development group using the online tool, Survey Monkey. All members of the group were sent a link to the online survey and asked to complete it. Each member was asked to rate their agreement with the statement ‘this measure is likely to be a good indicator of the performance of the emergency and urgent care system’, on a Likert scale anchored by 1 (‘disagree strongly’) and 9 (‘agree strongly’). There was also space for members to add any comments. Participants were asked not to limit their views about the potential usefulness of an indicator by perceived difficulties in collecting or processing the data required to calculate them. 14 consensus group members completed the survey.

A face to face meeting was held in October 2014 and all members of the consensus group who had completed the online survey were asked to attend. Thirteen of the 14 invited members attended the meeting. One member had planned to attend but was not able to do so because of a separate work commitment which arose close to the meeting. Each participant was provided with the original questionnaire which now included a record of their individual responses to the online survey and the group’s median score and interquartile ranges. The meeting was conducted using a nominal group technique format. Once each participant had been given the opportunity to provide their opinion about an indicator, that indicator was ranked again by the members of the group. This procedure was followed for each individual indicator until all indicators had been discussed. Following the meeting, the performance indicators were ranked by their median agreement score. Those with a median greater than 7 were classified as potentially good performance indicators. A second online survey was then created using the online tool, Survey Monkey. Those indicators which had scored a median greater than 7 were included and all participants were asked to rank these indicators in order of preference. Higher ranks indicate greater preference and are represented by lower numbers. This exercise was sent to the 13 members of the group who had attended the consensus development meeting and there was a 100% completion rate.

## Results

**Figure 1.**
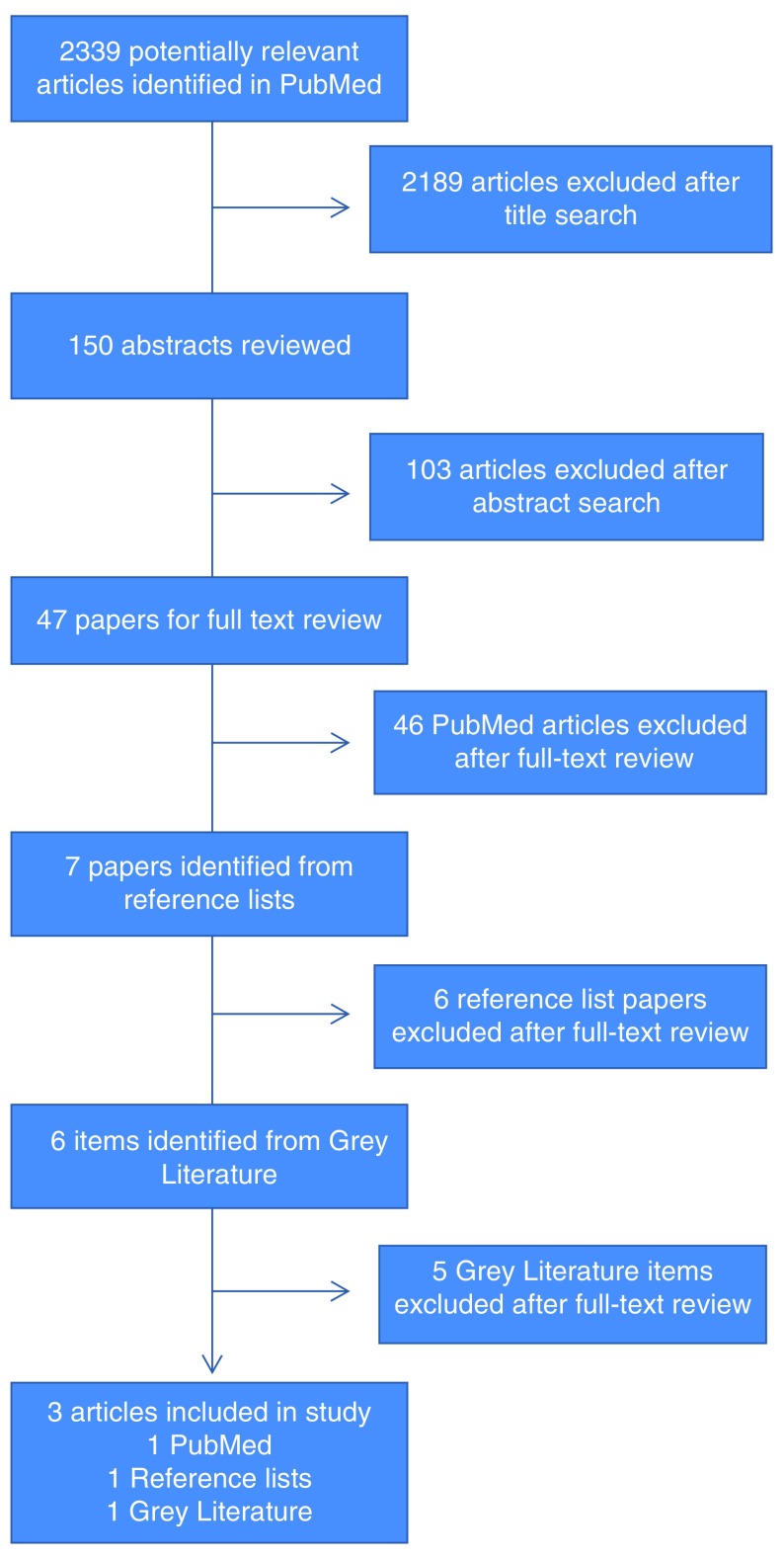
Flow chart of literature search.

The literature search strategy identified 2339 article titles. A title search reduced this to 150 articles and a review of the abstracts of these papers led to retrieval of 47 articles for a full-text review. A further seven articles were identified from the reference lists of the 47 full articles that were reviewed and six other documents from grey literature sources were selected for review. Two researchers reviewed the sixty items selected for full-text review (RD and JB). Following this review, fifty-seven were excluded for the following reasons: forty-four of the articles excluded at the full text stage were focused on service based indicators, seven reported on indicators that had been described by the previous systematic review and six were of a descriptive nature and not focused on specific indicators ( Figure 1). This process led to three articles being identified for inclusion in the review ^4, 6, 7^.

The three articles included in the final review yielded four novel indicators that had not been presented in the previous systematic review. These were: patient reported experience of whole episodes of emergency and urgent care ^4^; mortality rates among inter-hospital transfer patients ^6^; inter-hospital transfer times ^6^; and time from decision to admit to transfer to an appropriate inpatient bed ^7^. The combination of these new indicators with those that had been identified in the previous systematic review produced 42 unique indicators for review by the consensus development group. In total, 17 indicators had a median score on the Likert scale of agreement of greater than 7 following the consensus meeting (see Supplementary File 4). Table 1 presents the median, mean and range of rankings for these 17 indicators that were produced by the second online survey.

**Table 1.** Final ranking of 17 indicators likely to be useful indicators of the performance of Irish urgent and emergency care systems.

Median rank (range)	Mean rank	Performance indicator	Explanation
2 (1–10) *	3.9	Time from call to care for indicator conditions. E.g. for patients having thrombolysis, call to needle times. For patients having percutaneous coronary intervention (PCI), call to cath lab, for patients undergoing hip fracture repair, call to theatre	The aim of this indicator is to reduce times on patient journey through the EUCS to definitive care. It has been suggested that data for this indicator could be sourced from national and local recommendations for ‘definitive care’ for SEC and urgent conditions; service level linked data; ED data; AS patient report forms; and theatre books
3 (1–11)	4.4	Case fatality rates for serious, emergency conditions for which a well-performing EUCS could improve chances of survival	This indicator is based on health outcomes and it aims to reduce the proportion of patients with specified serious emergency conditions who die. This indicator could be calculated through HIPE and CSO Mortality Statistics.
7 (1–15)	7	Adherence to any evidence-based good practice guidelines for serious emergency, and urgent conditions	The aim of this indicator is to encourage services within EUCS to adopt good practice in managing patient care in accordance with the best available research evidence in published guidelines. This indicator would be measured through the auditing of practice and procedures that are implemented in EUCS.
7 (1–17)	7.5	Mortality rates among inter-hospital transfer patients for this group of conditions	This indicator aims to examine the best practice process of transfer of patients from one hospital to another and the mortality rates associated with this process. Data for this indicator may be collected by accessing both ambulance and HIPE data.
7 (3–16)	9.1	For EUCS users with the following group of serious, emergency conditions, who are admitted, the time from call to ambulance service to admission	This indicator aims to ensure that patients, who are admitted with serious emergency conditions, do so in an appropriate and timely manner. This data could be collected through HIPE, and ambulance data.
8 (2–16)	8.3	Call to ambulance service to time on scene.	This indicator aims to examine the variations in outcomes or processes due to differences in access and availability of care. A well performing EUCS will deliver or be working to deliver the same processes of care at all times and in all places. In order to measure this indicator, data sources required would include National Ambulance Service, GP records and Patient Surveys.
8 (1–17)	8.9	Patient reported experience of whole episodes of emergency and urgent care.	This can be measured through surveys similar to that which will be carried out as part of SIREN Work Package 4. The questionnaire addresses three domains of patient experience: entry into the system; progress through the system; and convenience of the system.
9 (2–15)	8.3	Time from decision to admit to transfer of patient to appropriate in-patient bed.	This indicator aims to ensure that patients who are admitted are appropriately placed in an in-patient bed in a timely manner. The data collected should include times of first contact, assessment and critical points in the patient’s journey. It could be measured through patient surveys and hospital audits.
9 (1–15)	8.9	Emergency re-admissions within 28 days as a proportion of all live discharges for the following group of urgent conditions	This indicator focuses on the processes within the emergency and urgent care system and its aim is to encourage services to work collaboratively in order to manage care both in hospital and in the post-discharge period. Data for this indicator could be sources from HIPE
9 (3–16)	10.4	For all of the serious emergency conditions combined, the proportion of deaths that occur before admission (i.e. in pre-hospital or in the Emergency Department)	This indicator aims to examine those patients with serious emergency conditions who die before admission to either pre-hospital or an ED. Data could be obtained from GPs
10 (3–16)	9.6	Hospital emergency admission rates for the following group of urgent conditions whose exacerbations could be managed out of hospital or in ED’s without admission to an inpatient bed	This focuses on avoidable admissions for acute exacerbations of urgent conditions. This indicator aims to reduce hospital admission rates for episodes that could be managed out of hospital or in settings without admission to a hospital bed.
11 (5–17)	10.9	Time from patient arrival at referring hospital to making the decision to transfer	This focuses on the processes associated with patient transfer to a hospital setting. It could be calculated using ambulance data
12 (1–17)	10.2	Case fatality rates for serious, emergency conditions for which a well-performing EUCS could improve chances of survival but for out of hospital deaths	This indicator is based on health outcomes and it aims to reduce the proportion of patients with specified serious emergency conditions who die outside of the hospital setting. Data could be obtained from GP’s and the coroner’s office.
12 (1–17)	10.6	Time from onset of serious emergency condition to arrival at the receiving hospital	This focuses on the processes associated with patient transfer to a hospital setting. It could be calculated through patient survey and ambulance data
12 (5–17)	12	Time from decision to transfer to availability of transport	This focuses on the processes associated with patient transfer to a hospital setting. It could be calculated using ambulance data
13 (4–17)	10.8	For EUCS users with the following group of serious, emergency conditions, who are admitted, the time from first contact with mental health team to admission	This indicator aims to ensure that patients, who are admitted with serious emergency conditions, do so in an appropriate and timely manner. This data could be collected through HIPE.
13 (4–17)	12.2	Arrivals at ED referred by any EUCS service and discharged without treatment or investigation(s) that needed hospital facilities	This indicator aims to enable EUCs to monitor the quality of the initial assessment and appropriate triage at the first point of contact. ED records could be used as the data source

*Higher ranks indicate greater preference and are represented by lower numbers.

## Discussion

Using a systematic review and nominal group consensus development exercise, we have identified a set of 17 indicators which a consensus group of different experts regard as potentially good measures of the performance of urgent and emergency care systems in Ireland. As far as we are aware this is only the second attempt to produce a set of indicators that are purely focused on system-level performance in urgent and emergency care and the first for Ireland. The indicator set was designed by Irish experts to be applicable to the Irish health system. Thus, it may not necessarily be applicable to health systems in other countries because of differences in terminology and how services are organised.

The list is made up of twelve process and five outcome indicators. Four of the seventeen indicators were included in the top sixteen indicators produced by a previous consensus development exercise carried out in the UK ^4^ and a further three were novel indicators which were identified through our systematic review. The three novel indicators were patient reported experience of whole episodes of emergency and urgent care ^4^; mortality rates among inter-hospital transfer patients ^6^; and time from decision to admit to transfer to an appropriate inpatient bed ^7^.

This study was undertaken using standard systematic review and consensus development methods. The members of the consensus group were purposively chosen as they were identified as having a wide range of expertise and knowledge in relation to various aspects of emergency and urgent care. The online survey allowed the opinions of those members to be collected and aggregated, while the face to face meeting offered the opportunity for the members to consider the indicators in light of hearing the opinion of their colleagues, as well as enabling discussion among panellists on the wording and clarity of the performance indicators.

### Study limitations

Our study has some limitations. The systematic review only covers the period up to 2014 and may, therefore, miss some more recent literature. Since 2014 at least two further consensus development studies have been published in this field. These studies were not focused on the whole system of urgent and emergency care: one covered only prehospital care ^8^ and one focused only on care in the ED ^9^. Nevertheless, it is notable that the study of prehospital performance found that “direct transport of ST-elevation myocardial infarction patients to a primary percutaneous intervention (PCI)-capable facility for ECG to PCI time <90 min” was the highest ranked indicator. This is consistent with our finding that ‘time from call to care’ for indicator conditions is rated as the best performance indicator for a whole system of urgent and emergency care. This provides some support to the validity of our consensus development exercise.

Data extraction in the systematic review was conducted independently by two reviewers who then came to consensus. However, no formal assessment of inter-rater reliability was conducted.

No attempt was made to achieve unanimity so it is possible that some of the indicators may be controversial to certain stakeholder groups. We also requested that panel members did not consider the feasibility of collecting data required to calculate an indicator. This may mean that the chosen performance indicators are not immediately measurable; however, we are hopeful that progress in data collection may allow these performance indicators to be measured in the future.

### Implications

The results of this paper have been used to conduct three studies on urgent and emergency care in Ireland, each using one of the highly ranked indicators described above. The first study measured “case fatality rates for serious, emergency conditions for which a well-performing EUCS could improve chances of survival” to assess whether national and regional population outcomes in Ireland over the period 2002–2014 were associated with the reconfiguration or regional urgent and emergency care systems. No distinct pattern of change was found among regions which underwent substantial reconfiguration compared to those that did not ^10^. The second study measured the impact of population and health system factors on county-level variation in “conditions whose exacerbations could be managed out of hospital or in ED’s without admission to an inpatient bed” in Ireland over the period 2014–2016 ^11^. This found that potentially avoidable emergency admissions are primarily driven by socioeconomic conditions, hospital admission policies and private health insurance coverage and were not associated with primary care resources. This is an important finding for policy in this area as it suggests that reductions in emergency admissions for ambulatory conditions will not necessarily be achieved by simply increasing the quantity of relevant primary care resources. The study also found that the distinction between ‘potentially avoidable’ and all other emergency admissions may not be as useful as previously believed when attempting to identify the causes of regional variation in emergency admission rates. The third study used “patient reported experience of whole episodes of emergency and urgent care” to compare user experiences of eight regional urgent and emergency care systems in Ireland ^12^. It found no consistent relationship between patient experience and the type of urgent and emergency care model in different regions, and concluded that composite questionnaire data may not offer a useful metric for exploring the impact of system-level service change.

Our research programme, including the present study, have produced some consistent learning points which may be useful for researchers in this field. First, it is not easy to distinguish between indicators of the performance of individual services and the system as a whole because of the inter-dependency of different services. For example, the indicator “call to ambulance time on scene” at first glance seems to cover only one service, but during the discussions of the consensus group it was felt to reflect a number of system-level issues such as the geographical configuration of ambulance stations and Emergency Department locations, and capacity pressures on the ambulance service that are caused by system-level decisions (e.g. need to spend more ambulance resources on inter-hospital transfers).

Second, it is difficult to operationalise research where populations, interventions and outcomes are difficult to define. For example, in all three of the studies described above the populations studied were compared at geographical levels (e.g. county, region) but these are inevitably an imperfect level of analysis because geographical units do not map perfectly to health system interventions, especially at boundaries where populations are exposed to both old and new models of care. This problem did not fatally undermine the internal validity of our case-fatality study ^10^ because all geographical units were subject to some ‘boundary leakage’ of populations, and the proportion of the overall study populations affected was low. In general, however, researchers should be sensitive to instances where this leakage is likely to pose a substantial challenge, for example when boundaries cut across urban areas and a high proportion of the population has easy access to more than one model of care.

A third, related issue, is the overall complexity of system evaluation. It is debatable whether traditional study designs are a useful way to compare whole systems of care because of the difficulty in making strong inferences about causal relationships within complex systems. Although there are growing calls for evaluations of complex organisational interventions at the level of whole health systems, progress has been limited because of the difficulty in dealing with complexity ^13^. In two of our studies ^10, 12^ we found no evidence of difference in outcomes between populations treated by different models of care. It is likely that true differences do exist for patient sub-groups on specific care pathways in particular contexts but the signal of these differences is hidden by system-level analyses for whole populations. Our studies provide useful information about aggregate outcomes and trends over time but more focused studies are required to provide evidence to policy makers about the impact of system changes such as hospital reconfiguration. These studies should focus on processes, such as compliance with new hospital referral guidelines, as well as outcomes ^13^. It is also possible that the impact of interventions in complex systems may not be apparent in the timeframes we used, and that longer-term studies are required ^13^.

Fourth, each indicator is targeted at a heterogeneous group of patients and is a composite of different outcomes. This can hide valuable information about patient sub-groups and specific outcomes. The case-fatality study, for example, combines outcomes for 16 different conditions ^10^. The patient experience study combined multiple questions into composite scale scores ^12^. Both of these found no signal of outcome differences between models of urgent and emergency care which may indicate that a finer grain of analysis is required when using performance indicators for urgent and emergency care systems.

Fifth, routinely collected secondary data can rarely be used to calculate the indicators presented in this paper and original data collection is often required. Routine data could only be used for two of the 17 indicators (case-fatality ratios for serious emergency conditions and admission rates for ambulatory conditions). The study of patient experience was original because no such data was available at the time it was conducted. Ireland has since introduced a national patient experience survey which is conducted annually ^14^. However, this still focuses on individual emergency departments and would not, therefore, be suitable for an analysis of the impact of whole system changes on patient experience. Time-based indicators are almost completely absent from the health information systems in public hospitals in Ireland.

Finally, one of our studies found that the indicator ‘emergency admission rates for conditions whose exacerbations could be managed out of hospital or in ED’s without admission to an inpatient bed’ was no more sensitive to variations in care settings than total emergency admission rates. This underlines the importance of a critical approach to performance indicators, which may not be fit for purpose despite seeming to have obvious face validity.

## Ethical statement

Ethical approval for the study was granted by the Clinical Research Ethics Committee of the Cork Teaching Hospitals [ECM 4 (q) 02/07/13]. The process of participants proceeding to the survey and completing it constituted consent.

## Data availability

The data is available on Open Science Framework: http://doi.org/10.17605/OSF.IO/3CW6F ^8^

Data are available under the terms of the Creative Commons Attribution 4.0 International license (CC-BY 4.0).

## Supplementary material

Supplementary File 1: PRISMA checklist.

.

Supplementary File 2: All novel indicators identified in the updated systematic review combined with those identified in the original systematic review.

.

Supplementary File 3: Definitions of urgent care-sensitive and emergency conditions.

.

Supplementary File 4: Median, range and mean of consensus development group ratings for all indicators following consensus development group meeting on a 9 point Likert scale. Higher scores represent a higher level of agreement that the indicator is a useful measure of system performance.
