# Supplementary material for: Development of performance indicators for systems of urgent and emergency care in the Republic of Ireland. Update of a systematic review and consensus development exercise
Source: HRB Open Res. 2019 Feb 12;1:6. Originally published 2018 Feb 28. [Version 2] doi: 10.12688/hrbopenres.12805.2 (PMC6973523; doi:10.12688/hrbopenres.12805.2)
Supplement: Supplementary file 3 [file hrbopenres-1-13970-s0002.tgz › 1cde019d-b9c5-44df-ad56-bb63fdf51cca.docx]

**Supplementary File 3: Definitions of urgent care-sensitive and emergency conditions**

Urgent care-sensitive conditions were defined as ‘conditions whose exacerbations could be managed by a well-performing EUC (emergency and urgent care system) out of hospital or in emergency departments (EDs) without admission to an inpatient bed.’

| **Serious, emergency conditions** | |
| --- | --- |
| 1 | Stroke/CVA |
| 2 | Myocardial Infarction |
| 3 | Major haemorrhage (for example, GI bleed, ruptured aneurysm) |
| 4 | Epilepsy |
| 5 | Diabetes |
| 6 | Appendicitis |
| 7 | Fractured neck of femur |
| 8 | Meningitis |
| 9 | Isolated extradural haematoma |
| 10 | Asthma |
| 11 | Septic Shock |
| 12 | Acute heart failure |
| 13 | Cardiac arrest |
| 14 | Exacerbation COPD |
| 15 | Anaphylaxis |
| 16 | Acute pancreatitis |
| 17 | Asphyxiation |
| 18 | Self-harm (suicide) |
| 19 | Falls |
| 20 | Assault |
| 21 | Burns |
| 22 | Road traffic crash injuries |
| 23 | Falls>65 Years |
| 24 | Poisoning |
| 25 | All external causes |

| **Urgent care-sensitive conditions** | |
| --- | --- |
| 1 | Angina |
| 2 | Non-specific chest pain |
| 3 | Asthma |
| 4 | COPD |
| 5 | Epileptic fit |
| 6 | Non-specific abdominal pain |
| 7 | Alcohol-induced coma |
| 8 | Drug overdose/poisoning |
| 9 | Hypoglycaemia |
| 10 | Minor head injuries |
| 11 | Syncope |
| 12 | Chest infection |
| 13 | Urinary tract infection |
| 14 | Chronic heart failure |
| 15 | Acute mental health crisis |
| 16 | Elderly falls |
