# Supplementary material for: Development of performance indicators for systems of urgent and emergency care in the Republic of Ireland. Update of a systematic review and consensus development exercise
Source: HRB Open Res. 2019 Feb 12;1:6. Originally published 2018 Feb 28. [Version 2] doi: 10.12688/hrbopenres.12805.2 (PMC6973523; doi:10.12688/hrbopenres.12805.2)
Supplement: Supplementary file 4 [file hrbopenres-1-13970-s0003.tgz › a47859f6-ffae-49b6-8c32-9fef864e811d_Supplementary_File_4_v2.docx]

## Supplementary File 4: Median, range and mean of consensus development group ratings for all indicators following consensus development group meeting on a 9 point Likert scale. Higher scores represent a higher level of agreement that the indicator is a useful measure of system performance.

|  | **Median (range)** | **Mean** | **Standard Deviation** |
| --- | --- | --- | --- |
| First person 999 contacts with the ambulance service who die within 3 days (first person contact is a patient who had contacted the service or for whom the service had been contacted) | 2 (1-7) | 3.4 | 2.3 |
| First person 999 contacts with the ambulance service who die within 7 days | 2 (1-7) | 3.0 | 2.2 |
| First person 999 contacts with the ambulance service who die within 3 days but limited to out of hospital deaths | 6 (1-8) | 4.7 | 2.8 |
| First person contacts (by phone/internet/in person) with any emergency and urgent care service, who die within 3 days (first person contact is a patient who had contacted the service or for whom the service had been contacted) | 2 (1-7) | 2.8 | 2.2 |
| First person contacts (by phone/internet/in person) with any emergency and urgent care service, who die within 7 days | 2 (1-6) | 2.6 | 1.9 |
| First person contacts (by phone/internet/in person) who die within 3 days but limited to out of hospital deaths | 4.5 (1-8) | 4.2 | 2.9 |
| Mortality rates for serious, emergency conditions in the population aged <70 years old, for which a well-performing EUCS could improve chances of survival | 7 (5-9) | 7.3 | 1.2 |
| Mortality rates for serious, emergency conditions in the population aged <70 years old, for which a well-performing EUCS could improve chances of survival but for those who die out of hospital | 6.5 (1-9) | 6.1 | 2.4 |
| Case fatality rates for serious, emergency conditions for which a well-performing EUCS could improve chances of survival | 8 (4-9) | 7.3 | 1.3 |
| Case fatality rates for serious, emergency conditions for which a well-performing EUCS could improve chances of survival but for out of hospital deaths | 7.5 (2-9) | 6.8 | 2.1 |
| For all of the serious emergency conditions listed below combined, the proportion of deaths that occur before admission (i.e. in pre-hospital or in the Emergency Department). Stroke/CVA, Myocardial Infarction, Major haemorrhage (for example GI bleed or ruptured aneurysm), Epilepsy, Diabetes, Cholecystitis, Appendicitis, Fractured neck of femur, Meningitis, Isolated extradural haematoma, Asthma, Septic Shock, Acute heart failure, Cardiac arrest, Exacerbation COPD, Anaphylaxis, Acute pancreatitis, Asphyxiation, Self-harm (suicide), Falls, Assault, Burns, Road traffic crash injuries, Falls>65, Poisoning, All external causes. | 8 (4-9) | 7.3 | 1.4 |
| Patient reported experience of whole episodes of emergency and urgent care | 8 (6-9) | 7.6 | 0.9 |
| Mortality rates among inter-hospital transfer patients for this group of conditions: Stroke/CVA, Myocardial Infarction, Major haemorrhage (for example GI bleed or ruptured aneurysm), Epilepsy, Diabetes, Cholecystitis, Appendicitis, Fractured neck of femur, Meningitis, Isolated extradural haematoma, Asthma, Septic Shock, Acute heart failure, Cardiac arrest, Exacerbation COPD, Anaphylaxis, Acute pancreatitis, Asphyxiation, Self-harm (suicide), Falls, Assault, Burns, Road traffic crash injuries, Falls>65, Poisoning, All external causes) | 8 (5-9) | 7.7 | 1.2 |
| Numbers of hospital emergency inpatient admissions discharged in <24 hours as proportion of all emergency admissions | 6 (1-9) | 6.1 | 2.3 |
| Numbers of hospital emergency inpatient admissions discharged in <72 hours as proportion of all emergency admissions | 6 (1-9) | 5.5 | 2.5 |
| Hospital emergency admission rates for the following group of urgent conditions whose exacerbations could be managed out of hospital or in ED's without admission to an inpatient bed: Angina, Non-specific chest pain, Asthma, COPD, Epileptic fit, Non-specific abdominal pain, Alcohol induced coma, Drug overdose/poisoning, Hypoglycaemia, Minor head injuries, Syncope, Chest infection, Urinary tract infection, Chronic heart failure, Acute mental health crisis, Elderly falls) | 8 (6-8) | 7.5 | 0.7 |
| Proportion of contacts with EUCS services resulting in a hospital inpatient admission for the following group of urgent conditions: Angina, Non-specific chest pain, Asthma, COPD, Epileptic fit, Non-specific abdominal pain, Alcohol induced coma, Drug overdose/poisoning, Hypoglycaemia, Minor head injuries, Syncope, Chest infection, Urinary tract infection, Chronic heart failure, Acute mental health crisis, Elderly falls | 6 (1-9) | 5.6 | 2.2 |
| Emergency re-admissions within 28 days as a proportion of all live discharges for the following group of urgent conditions: Angina, Non-specific chest pain, Asthma, COPD, Epileptic fit, Non-specific abdominal pain, Alcohol induced coma, Drug overdose/poisoning, Hypoglycaemia, Minor head injuries, Syncope, Chest infection, Urinary tract infection, Chronic heart failure, Acute mental health crisis, Elderly falls) | 8 (5-9) | 7.5 | 1.1 |
| Adherence to any evidence-based good practice guidelines for serious emergency, and urgent conditions | 8 (2-9) | 7.3 | 1.9 |
| Rate of frequent attendees at ED per 1000 population, standardised for distance from the ED | 7 (3-8) | 6.7 | 1.4 |
| Rate of frequent attendees at ED as a proportion of all ED attendees, standardised for distance from the ED | 7 (3-8) | 6.6 | 1.5 |
| Arrivals at ED by emergency ambulance and discharged without treatment or investigation(s) that needed hospital facilities | 7 (1-9) | 6.4 | 2.3 |
| Arrivals at ED referred by any EUCS service and discharged without treatment or investigation(s) that needed hospital facilities | 8 (5-9) | 7.4 | 1.3 |
| Self-referred re-attendance at urgent ambulatory care services (ED's, out of hours care, minor injury unit, mental health crisis team etc.) within 7 days of initial presentation to any EUCS service. | 7 (1-9) | 6.5 | 1.9 |
| Total calls to emergency ambulance service for transfer of patients between EUCS services | 7 (3-9) | 7.1 | 1.5 |
| Total attendance at EUCS services referred elsewhere for primary management | 7 (1-9) | 5.8 | 2.3 |
| Multiple transfers between EUCS services | 7 (1-8) | 6.4 | 2.1 |
| Patients with emergency or urgent conditions who are referred from one service to another who do not subsequently attend | 5 (1-8) | 5.3 | 2.0 |
| Call to ambulance service to time on scene | 8 (6-9) | 8.0 | 0.8 |
| First contact with Out of Hours service to clinical assessment (by nurse, Emergency Care Practitioner, GP etc.) | 7 (1-9) | 6.6 | 2.4 |
| From call to ambulance service to admission | 8 (3-9) | 7.2 | 1.6 |
| First contact with Out of Hours service to admission | 7 (1-8) | 6.2 | 2.4 |
| First contact with mental health team to admission | 8 (2-8) | 6.6 | 2.1 |
| Time from call to care for indicator conditions. For example, for patients having thrombolysis, call to needle times. For patients having percutaneous coronary intervention (PCI), call to cath. lab, For patients undergoing hip fracture repair, call to theatre | 9 (6-9) | 8.4 | 0.9 |
| Time from decision to admit to transfer of patient to an appropriate in-patient bed | 8 (1-9) | 7.5 | 2.5 |
| Time from patient arrival at referring hospital to making the decision to transfer | 8 (3-9) | 8.0 | 1.6 |
| Time from decision to transfer to availability of transport | 8 (7-9) | 8.4 | 0.7 |
| Time taken to transfer from referring hospital to receiving hospital | 7 (1-9) | 6.8 | 2.2 |
| Time from onset of serious emergency condition to arrival at the receiving hospital | 8 (1-9) | 7.3 | 2.4 |
| Proportion of population living within 10km of emergency and urgent ambulatory care facilities (ED's, out of hours care, minor injury unit, mental health crisis team etc.) | 6 (2-9) | 5.8 | 2.0 |
| Proportion of population living within 10km of 24 hour emergency and urgent ambulatory care facilities (ED's, out of hours care, minor injury unit, mental health crisis team etc.) | 6 (2-9) | 5.7 | 2.3 |
