# Supplementary material for: Development of performance indicators for systems of urgent and emergency care in the Republic of Ireland. Update of a systematic review and consensus development exercise
Source: HRB Open Res. 2019 Feb 12;1:6. Originally published 2018 Feb 28. [Version 2] doi: 10.12688/hrbopenres.12805.2 (PMC6973523; doi:10.12688/hrbopenres.12805.2)
Supplement: Peer review history

# Peer review: Reviewer response for version 2

**DOI:** 10.21956/hrbopenres.13970.r26619

**Published:** 2019-06-26

**Authors:** Anna Cantrell

**Affiliations:** 1 School for Health and Related Research (ScHARR), University of Sheffield, Sheffield, UK

The abstract contains all of the relevant information. The results section description of the literature flow chart does not agree with the flow chart of the literature review, more detail below.

 The introduction section provides a clear introduction to the topic.

 The methods for the systematic review are clear. It might have been useful for the authors to search another database in addition to Medline, perhaps Embase, Cochrane Library, CINAHL or HMIC but the decision to just search Medline might have been due to time, resource and access constraints. It would have been useful to include the PubMed search strategy in format that would be reproducible, this could be as supplementary material if there is not space in the article. The sources contacted for grey literature are appropriate and the search for and inclusion of grey literature is a strength of the review. I am not concerned by the dates of the search as the articles retrieved were used to develop a list of novel indicators for the consensus development exercise which has now occurred and it is discussed as a limitation that the systematic review only covers up to 2014 and more recent literature could have been missed.

 The inclusion of the PRISMA checklist is useful but I don't think that the information about the search strategy is sufficient to enable it to be repeated. The PRISMA checklist states "Present full electronic search strategy for at least one database", this article only includes the statement by combining variants of the text terms 'emergency' and 'indicator' using the AND operator. 

 The methods of the consensus development exercise are again clear. There is detailed information about the broad range of experts involved. 

 The results section first describes the results of the literature search and is reasonably clear but hard to follow in conjunction with the flow chart. The flow chart for the literature review is difficult to follow and hard to correlate with the results section in the abstract. For example, the chart has the 2339 articles from the literature search then the results sections mentions further searches and then 60 items were identified for full-text review but it is 47 in the flow chart. Completing The PRISMA 2009 Flow Diagram from here would have been more appropriate and then hopefully clearer. The results of the consensus development exercise are clear.

 The study limitation section is appropriate, all the limitation mention are relevant and described sufficiently.

 The implications section is well organised starting by discussing three studies that have been been conducted using the results of this article then the implications for research.

 Overall, a good interesting paper.

I confirm that I have read this submission and believe that I have an appropriate level of expertise to confirm that it is of an acceptable scientific standard.

# Peer review: Reviewer response for version 2

**DOI:** 10.21956/hrbopenres.13970.r26625

**Published:** 2019-06-11

**Authors:** Maaret Castrèn

**Affiliations:** 1 Department of Emergency Medicine and Services, Helsinki University Hospital and Helsinki University, Helsinki, Finland

After reading the reviewers reports I still have some concerns. It would have benefited the article if the search would have been done for more new articles, 2014 seems like a long time ago. And even if you explain this later, the reader sees 2014 as the deadline.

 You state that you exclude individual components but still you have some in the indicators so this remains unclear for the reader. You never really explain what happened to the 6 grey literature other documents.

 Figure 1 should be written in a different format, now you have it like the flow would be from the 2339 potentially relevant articles and of course this is not the case so you need to make three different flows in this figure.

 It is not clear in Table 1 what EUCS is so write it out and also SIREN work package is unclear for the reader. I don’t understand why you include inter-hospital transfers, most of them being not acute or emergency medicine.

I confirm that I have read this submission and believe that I have an appropriate level of expertise to confirm that it is of an acceptable scientific standard, however I have significant reservations, as outlined above.

# Peer review: Reviewer response for version 2

**DOI:** 10.21956/hrbopenres.13970.r26511

**Published:** 2019-03-15

**Authors:** Robbert Huijsman

**Affiliations:** 1 Institute of Health Policy and Management, Erasmus University Rotterdam, Rotterdam, The Netherlands

I propose to accept the paper, although it is a pity that the authors did not follow the suggestion of both reviewers to extend the literature search which now stops in 2014 (5 years ago!).

I confirm that I have read this submission and believe that I have an appropriate level of expertise to confirm that it is of an acceptable scientific standard.

# Peer review: Reviewer response for version 1

**DOI:** 10.21956/hrbopenres.13865.r26092

**Published:** 2018-05-01

**Authors:** Ellen Weber

**Affiliations:** 1 Department of Emergency Medicine, University of California, San Francisco, San Francisco, CA, USA

Thank you for the opportunity to review this paper. It is nicely written and succinct. The research question is important and novel; there have several studies in the field of prehospital care and emergency medicine looking at performance indicators but they usually focus on service in the system. The idea of finding indicators that reflect the performance of the whole system is important and could lead to alignment of incentives that currently do not exist, in both Ireland and beyond. Overall the study is well-done. I have a few concerns about a few of the indicators representing (whole system) see later, but overall, the indicators seem to represent the goals the authors set out to achieve and many have not been emphasized previously.

 I have several suggestions to strengthen the paper.  First, the systematic review only goes to 2014. Since then there have been at least two other Delphi studies on indicators in emergency medicine ^1^^,^^2^.  This is a limitation if the only indicators assessed were those found in these reviews (i.e. none submitted by the experts). Although I understand that the study rests on what was available at the time, I would improve the strength of the paper if the authors could do an updated literature review and see if there are any other indicators that might have been included had these newer articles been in the systematic review.  Additionally, it would helpful if the authors could summarise the newly added articles in a list in an appendix with articles, dates, authors, location, service(s) involved, etc.

 2). The methods by which the articles and the performance indicators were initially chosen is not well described. How many individuals were involved in the article selection, and how was the process of extracting the new indicators from the articles done. Was there any cross-checking among the investigators? I would imagine that there would be some areas of judgment here as to whether an indicator was new or differently stated, as well as whether it applied to the whole system. Was there any type of inter-rater reliability assessed?

 3) The discussion should be expanded to include comparisons of this work with prior processes within emergency medicine to identify key performance indicators. You have a brief mention of the UK exercise, although this comes a bit too early in the discussion, and without much detail. (You also say that 6 were from your systematic review but you originally identified only 3 new ones compared with the prior review..) It would help to point out what indicators were novel, which part of the systematic review they originated in, and why you think your indicators were different.  It would also help to a discussion of the pragmatic implications of this work – how have such performance indicators been use to evaluate systems of care and how do the authors imagine theirs being used in the context of Ireland’s reconfiguration.

 A few other points that need clarity:

  

1. The description of the article search and selection does not readily match the flow diagram. EG the flow diagram doesn’t show the 150 articles that had abstracts read. And then the 47 articles that were reviewed.
2. How was the grey literature identified?
3. Were all experts from Ireland?
4. There were 17 experts in the total group, and you say that 13/14 attended the meeting. What happened to the other 3?
5. Could you explain the procedure at the meeting a bit more – did each of the 13 participants speak about each of the indicators. (Sounds like a very long day!)  What is meant by a “nominal” group technique.
6. The lists of indicators both in the main text and the appendix are confusing. In Table 1 you have 17 indicators which you said had at least a median score of 7 in the meeting and they were then ranked in an on-line process. It is not clear how those with a median of 2 and 3 are included. I am also not sure the reader needs to see the “mean rank” but rather the final ranking order based on all responses. Even if you keep those ranks in, it would help to organize the table by ranking. Additionally, you could include a notation for which are process and outcome measures, and which (if any) were from the updated systematic review.  In the appendix, its not clear you need to have two separate lists of all measures. Could you combine them, and present them according to type (process, structure, outcome).
7. I wonder if the authors would also care to discuss some of the indicators which don’t seem to this reviewer to represent “whole system.” Performance.  E.g. Call to ambulance to time on scene. While this is described as being useful to measure variation, it is still very “service” specific – regarding the prehospital system. Similar question for “Time from onset of serious emergency condition to arrival at the receiving hospital”.
8. Abstract – The methods of the abstract should be expanded to explain a bit more about the updating of a prior review, and more about the consensus exercise, including the variety of experts. Provide a brief explanation of “nominal”.

Minor editorial issues:

 Page 3, third paragraph – the last sentence –  “This variation represents….” Is probably unnecessary and takes the introduction a bit off topic.

 Page 3, first sentence of methods – Please add the publication date of the first systematic review to the text so readers don’t have to search your references.

 Page 4, second paragraph – should be indicators

 Page, 4 end of third paragraph, the” This review led to three articles being identified for inclusion in the review” - might change to: This process led to …

I confirm that I have read this submission and believe that I have an appropriate level of expertise to confirm that it is of an acceptable scientific standard, however I have significant reservations, as outlined above.

## References

[1] : Development of key performance indicators for prehospital emergency care. *Emerg Med J*.2016;33(4) : 10.1136/emermed-2015-204793 286-92 10.1136/emermed-2015-204793 26796739

[2] : Developing Emergency Room Key Performance Indicators: What to Measure and Why Should We Measure It?. 10.3233/978-1-61499-664-4-179 179-182 10.3233/978-1-61499-664-4-179 27350498

# Peer review: Reviewer response for version 1

**DOI:** 10.21956/hrbopenres.13865.r26158

**Published:** 2018-04-23

**Authors:** Robbert Huijsman

**Affiliations:** 1 Institute of Health Policy and Management, Erasmus University Rotterdam, Rotterdam, The Netherlands

Please add in subtitle that it's an update of a systematic review. How did you verify your results with those of the authors of the previous review (and for instance check your decision to exclude 7 papers from that review). Your update is already slightly outdated, because you performed it in August 2014. Although you were quick with the consensus group meeting in October 2014, directly after your review update, now it is more than 3 years later. Please reflect on this in your Discussion, especially as the acute field is evolving rapidly. Also discuss the generalisability of your findings for Ireland to other countries with different systems of emergency and acute care.

 The number of experts in your consensus group goes down in two steps from 17 (bottom line on page 2), to 14 and then 13. Please explain in more detail the reasons for dropout in two steps, and possible impact on your findings.

 In the Results, your text about excluded articles does not parallel the semi-final box in Figure 1, please synchronize. The way of scoring is not totally clear to me. How did the ranking actually take place and what then is the meaning of median scores and the differing score ranges per indicator? Your threshold of 7 has a different meaning if indicators vary in their range, so the order in the table is not that self evident. Experts ranked the indicators twice, it seems. In the first round you selected indicators with a median higher than 7, but how then can Table 1 about the second round of ranking include two indicators with a median of (much) lower than 7? You added * and ** to some entries in Table 1, but their is no explanation below the table.

 Your Discussion is rather short and poor, please elaborate more on the content of your findings, in combination with the existing literature and professional guidelines, not just restrict yourselves to a (too short) discussion on the methods. For instance, your recommendations on future steps about measurement are rather superficial. Perhaps reflect on a Registry, a quality system with feedback and improvement cycles, or other ways to incorporate performance indicators in the real work of professionals and managers in emergency care.

I confirm that I have read this submission and believe that I have an appropriate level of expertise to confirm that it is of an acceptable scientific standard, however I have significant reservations, as outlined above.
